# Evaluation of the Biogenic Amines Formation and Degradation Abilities of *Lactobacillus curvatus* From Chinese Bacon

**DOI:** 10.3389/fmicb.2018.01015

**Published:** 2018-05-15

**Authors:** Lu Li, Xiaoxue Wen, Zhiyou Wen, Shouwen Chen, Ling Wang, Xuetuan Wei

**Affiliations:** ^1^Key Laboratory of Environment Correlative Dietology (Ministry of Education), College of Food Science and Technology, Huazhong Agricultural University, Wuhan, China; ^2^State Key Laboratory of Agricultural Microbiology, Huazhong Agricultural University, Wuhan, China; ^3^Department of Food Science and Human Nutrition, Iowa State University, Ames, IA, United States; ^4^Hubei Collaborative Innovation Center for Green Transformation of Bio-Resources, College of Life Sciences, Hubei University, Wuhan, China

**Keywords:** biogenic amines, formation, degradation, *Lactobacillus cxurvatus*, Chinese bacon

## Abstract

Control of biogenic amines (BAs) is critical to guarantee the safety of fermented meat products. The aim of this study is to evaluate the BAs formation and degradation abilities of lactic acid bacteria from Chinese bacon to obtain the beneficial candidate for BAs control. Seven lactic acid bacteria were selected from the typical Chinese bacon products, identified as *Lactobacillus curvatus* by 16S rDNA analysis. Then, genes analysis and high-performance liquid chromatography (HPLC) analysis were performed to evaluate the BAs formation and degradation abilities of as-selected strains. All *L. curvatus* strains were confirmed to harbor the genes encoding the tyrosine decarboxylase and ornithine decarboxylase, and they could produce tyramine, β-phenethylamine, putrescine, and cadaverine. In comparison, the lowest concentration of total BAs was obtained in *L. curvatus* G-1. Meanwhile, all *L. curvatus* strains were positive in amines oxidase gene analysis, and they could also degrade six common BAs, especially the *L. curvatus* G-1 with the highest degradation percentage (above 40%) for each BA. Furthermore, fermented meat model analysis verified that the *L. curvatus* G-1 could significantly reduce BAs. In conclusion, *L. curvatus* G-1 shows a low BAs-producing ability, as well as a high BAs-degrading ability, and this study provides a promising candidate for potential BAs control in fermented meat products.

## Introduction

Biogenic amines (BAs), a group of low-molecular-weight nitrogenous compounds, are mainly produced through amino acid decarboxylation, which exists in most fermented foods such as cheese, yoghurt, sausage, wine, and beer (Landete et al., 2005; Lázaro et al., 2015; Guarcello et al., 2016). The BAs reported in fermented foods include histamine, tyramine, tryptamine, putrescine, cadaverine, and phenylethylamine, which are generated by decarboxylation of histidine, tyrosine, tryptophan, ornithine, lysine, and phenylalanine, respectively (Landete et al., 2007b; Romano et al., 2013; Diaz et al., 2015; Gardini et al., 2016). Moreover, putrescine can also be formed through deimination of agmatine (Coton et al., 2010). Low concentrations of BAs are essential for normal metabolic functions in animals, plants, and microorganisms. However, the excessive oral intake of BAs can lead to nausea, headache, rashes, and blood pressure changes (Ladero et al., 2010). Therefore, control of BAs within the safety level is critical for the safety of fermented foods.

Biogenic amines have been broadly reported as potentially toxic substances in fermented meat products (Lu et al., 2010; Zhang et al., 2013; Nie et al., 2014; Wang et al., 2015a,b). Lactic acid bacteria are dominant in fermented meat products and involved in the formation of color, texture, and flavor (De Martinis and Freitas, 2003; Cornu et al., 2011). However, there is also a safe risk from some lactic acid bacteria due to their excessive BAs accumulation abilities (Suzzi and Gardini, 2003). Microbial decarboxylases are responsible for forming BAs (Nie et al., 2014). As the starter cultures, lactic acid bacteria can hydrolyze meat proteins to release free amino acids (Silvina et al., 2010), and their decarboxylases can further catalyze amino acids to BAs (Suzzi and Gardini, 2003). On the other hand, some lactic acid bacteria can synthesize amine oxidases to degrade BAs (Dapkevicius et al., 2000; García-Ruiz et al., 2011). Different strains usually have different BAs formation and degradation abilities. Previous reports focused on screening of the BAs-degrading strains to reduce the BAs contents (Latorre-Moratalla et al., 2010; García-Ruiz et al., 2011; Cueva et al., 2012; Zaman et al., 2014), while only few reports aimed to select the strain with high BAs-degrading ability as well as low BAs-producing activity (Herrerofresno et al., 2012; Zhang et al., 2013), which will be more beneficial to control the BAs.

Chinese bacon products, such as Guangdong, Sichuan, and Hunan bacon, are the traditional and popular fermented meat products due to their unique flavor and texture (Huang et al., 2014). Chinese bacon products contain rich lactic acid bacteria (Liu et al., 2011), while no report evaluates their BAs formation and degradation abilities to screen the BAs-controlling bacteria. Therefore, screening the low BAs-productive lactic acid bacteria with high BAs-degrading ability from Chinese bacon products may provide novel candidates for BAs control.

Qualitative and quantitative tests of BAs have been broadly reported. High-performance liquid chromatography (HPLC) analysis can acquire reliable qualitative and quantitative data of individual amines (Ordóñez et al., 2016). In addition, the polymerase chain reaction (PCR) possesses the advantages of rapidity, sensitivity, and specificity to detect decarboxylase genes to indicate the potential BAs risk, and several research groups have utilized PCR technique to detect the BAs-producing lactic acid bacteria (Elsanhoty and Ramadan, 2016; Shiling et al., 2016). Therefore, the aim of this study is to evaluate the BAs formation and degradation abilities of lactic acid bacteria from Chinese bacon through PCR and HPLC analysis and then to select the beneficial candidate with low BAs formation and high BAs degradation ability for BAs control in fermented meat products.

## Materials and Methods

### Samples and Chemicals

Bacon samples were obtained from the supermarkets of Chengdu (Sichuan), Hunan (Changsha), Hubei (Wuhan), Guangdong (Guangzhou), Shanxi (Xian), and Gansu (Lanzhou) of China at the March of 2017. All samples were collected in three replicates, and stored at -20°C. The standard substances (histamine, tyramine, tryptamine, β-phenethylamine, putrescine, cadaverine, and 1, 7-dimethylheptane) were purchased from Sigma–Aldrich Co., LLC (St. Louis, MO, United States). Other chemicals were bought from Sinopharm Chemical Reagent Co., Ltd. (Shanghai, China).

### Isolation of Lactic Acid Bacteria

Bacon samples (5 g) were crushed and added into 45 mL sterile water and rotated at 37°C and 140 rpm for 40 min. The liquid mixtures were diluted, spread onto the solid MRS medium (peptone 10 g/L, beef extract 8 g/L, yeast extract 4 g/L, glucose 20 g/L, diammonium hydrogen citrate 2 g/L, sodium acetate 5 g/L, K_2_HPO_4_ 2 g/L, MgSO_4_ 0.2 g/L, MnSO_4_ 0.04 g/L, Tween 80 1 g/L, agar 14 g/L, pH = 5.7) added with 20 g/L CaCO_3_, and cultured at 37°C for 24 h in a vacuum bag. The single colony with the big dissolved calcium zone was selected as the potential lactic acid bacterium.

### Identification of Strains

Genomic DNA of lactic acid bacteria was extracted using Gen-EluteTM Kit (Tiangen Biotech Co., Ltd, Beijing, China) according to the manufacturer’s instructions, and the DNA concentration and quality were estimated on a Nano Drop2000 spectrophotometer (Thermo, United States). The 16S rDNA sequence analysis was carried out according to our previous report (Wei et al., 2011). The 16S rDNA sequence was amplified using the universal primers of 27f (AGAGTTTGATCMTGGCTCAG) and 1492r (CTACGGCTACCTTGTTACGA), following the PCR procedure: 95°C for 5 min; 95°C for 45 s, 55°C for 1 min, 72°C for 1 min, 32 cycles; 72°C for 10 min; and 4°C for 10 min. PCR is performed in 25 μL reaction mixture containing 2 μL template DNA, 2.5 μL Easy-Taq Buffer, 2.5 μL dNTPs, 1 μL of each primer, 0.3 μL Easy-Taq enzyme, and 15.7 μL nuclease-free water. The PCR products were purified, recovered, and sequenced by the Tsingke Biological Technology Co., Ltd. (Wuhan, China). The sequence similarity analysis was carried out by the Blastn program^1^, and the phylogenetic tree was generated using the MEGA 5.0.

### Detection of the Genes Related to BAs

Polymerase chain reaction amplification and sequence analysis were performed to confirm the presence of BAs-related genes of histidine decarboxylase (*hdcA*), tyrosine decarboxylase (*tyrdc*), ornithine decarboxylase (*odc*), agmatine deiminase (*aguA* and *aguD*), lysine decarboxylase (*ldc*), and multi-copper oxidase (*sufI*). The primers used in this study were listed in **Table 1**. PCR amplification is performed in 25 μL reaction mixture containing 2 μL of template DNA, 2.5 μL Easy-Taq Buffer, 2.5 μL dNTPs, 1 μL of each primer, and 0.3 μL Easy-Taq enzyme wherein nuclease-free water was added to reach 25 μL. The reactions were performed according to the parameters shown in **Table 2**. The PCR products were analyzed by electrophoresis on a 0.8% agarose gel and revealed under UV after staining with ethidium bromide. And the PCR amplicons were purified, recovered, and gene sequencing was performed by the Tsingke Biological Technology Co., Ltd. (Wuhan, China). The sequence similarity analysis was conducted through the Blastn program^2^.

**Table 1:** Lists of primers to detect the genes involved in the formation and degradation of BAs.

Enzyme	Primer name	Sequence 5′–3′	Expected amplicon size	Reference
Histidine	HdC1	TTGACCGTATCTCAGTGAGTCCAT	174 bp	Fernández et al., 2006
decarboxylase	HdC2	ACGGTCATACGAAACAATACCATC		
Tyrosine	TD2	ACATAGTCAACCATGTTGAA	1100 bp	Coton et al., 2004
decarboxylase	TD5	CAAATGGAAGAAGAAGTAGG		
Ornithine	ODF	CATCAAGGTGGACAATATTTCCG	500 bp	Elsanhoty and Ramadan, 2016
decarboxylase	ODR	CCGTTCAACAACTTGTTTGGCA		
Agmatine	AgmSq1	CAAGATTTDTTCTGGGCHTTYTTCTC	700 bp	Ladero et al., 2011
deiminase	AgmSq2	TTGGHCCACARTCACGAACCCT		
Agmatine	AgD1	CAYGTNGAYGGHSAAGG	600 bp	Coton et al., 2010
deiminase	AgD2	TGTTGNGTRATRCAGTGAAT		
Lysine	Cad2F	CAYRTNCCNGGNCAYAA	1185 bp	Landete et al., 2007a
decarboxylase	Cad2R	GGDATNCCNGGNGGRTA		
Multi-copper	BCf	CAGGGGATGGACGAAGGTGT	329 bp	Guarcello et al., 2016
oxidase	BCr	TCTTGCTTTGGCTTGGCTGG		

**Table 2:** PCR protocol of each gene.

Primer pair	Initial denaturation	Denaturation	Annealing	Extension	Cycles
HdC1/HdC2	94°C, 2 min	94°C, 30 s	52°C, 40 s	72°C, 30 s	35
TD2/TD5	95°C, 5 min	95°C, 45 s	52°C, 30 s	72°C, 1 min	31
ODF/ODR	95°C, 5 min	95°C, 45 s	52°C, 30 s	72°C, 1 min	31
AgmSq1/AgmSq2	94°C, 2 min	94°C, 30 s	52°C, 30 s	72°C, 1 min	30
AgD1/AgD2	94°C, 2 min	94°C, 30 s	52°C, 30 s	72°C, 1 min	30
Cad2F/Cad2R	94°C, 2 min	94°C, 30 s	52°C, 30 s	72°C, 1 min 30 s	30
BCf/BCr	95°C, 5 min	95°C, 30 s	58°C, 30 s	72°C, 1 min 30 s	31

### Evaluation of BAs-Producing and Degradation Ability

To evaluate the BAs-producing ability, as-selected strains were inoculated into the MRS broth, respectively, incubated statically at 37°C for 24 h as the seed cultures, which were further transferred into 5 mL MRS broth supplemented with 1 g/L of histidine, tyrosine, tryptophan, phenylalanine, ornithine monohydrochloride, lysine, or agmatine sulfate salt. After static culturing at 37°C for 48 h, the concentrations of BAs were determined. For the BAs-degrading ability analysis, the 24 h cultures of each strain were harvested by centrifugation at 6000 ×*g* for 10 min, and the cell pellets were washed with 0.05 M phosphate buffer (pH = 7) and re-suspended (OD_650_ = 0.4) in 0.05 mol/L phosphate buffer consisting of 100 mg/L of histamine, tyramine, tryptamine, β-phenethylamine, putrescine, and cadaverine. The phosphate buffer without inoculation of *Lactobacillus curvatus* was used as the control. After static incubation at 37°C for 24 h, the concentrations of BAs were determined. The value of BA-degradation rate was determined based on an equation, i.e., *M* = [(*A*-*B*)/*A*] × 100%, where *M* is the percentage of BAs degradation, and A and B represent the initial BAs content and residual BAs content, respectively.

### Fermented Meat Product Model Analysis

The fermented meat model was constructed to evaluate the BAs-controlling abilities of selected strains. The cells were inoculated into the MRS broth, statically cultured at 37°C for 24 h as the liquid seed cultures (OD_650_ = 0.8), which were then transferred (3%, v/w) into the fresh pork slices (10 g) added with 2% salt, 1% glucose, and 4% sucrose in sterilized flasks (50 mL). The cells were inoculated into the MRS broth, statically cultured at 37°C for 24 h as the seed cultures, which were then transferred into 10 g fresh pork slices containing 2% salt, 1% glucose, and 4% sucrose in sterilized flasks (50 mL). Thereafter, the mixtures were incubated at 37°C for 7 days, and the total BAs concentration of each sample was analyzed by HPLC. The fermented experiments were performed by inoculation with *L. curvatus* G-1 and *L. curvatus* B-1, respectively, as well as mixed *L. curvatus* G-1 and *L. curvatus* B-1, and the sample inoculated with equal volume of sterilized water was used as the control.

### Determination of BAs

Analysis of BAs was performed according to a previous study (Kim et al., 2012). Briefly, to extract the BAs, 0.5 g solid sample or 0.5 mL liquid sample was mixed with 1.5 mL of 0.4 M HClO_4_ for 1 h. The extracts were centrifugated at 12,000 ×*g* for 10 min to collect the supernatant (250 μL), which was mixed with 25 μL of NaOH (2 M) and 75 μL of saturated NaHCO_3_ and then reacted at 50°C for 45 min after supplement with 500 μL of dansyl chloride (5 mg/mL). Then, 25 μL of NH_4_OH (25%) was added into the reactant to remove the residual dansyl chloride, and the final volume was increased to 1.5 mL with acetonitrile. The final reaction system was centrifuged at 2500 ×*g* for 5 min to obtain the supernatant, which was filtered by a 0.22 μm membrane for further analysis. The HPLC analysis was operated on an Agilent 1260 system under the parameters of Zorbax Eclipse XDB-C18 (4.6 mm × 250 mm, 5 μm), column temperature (30°C), flow rate (1 mL/min), and detection wavelength (254 nm). Using acetonitrile and H_2_O as mobile phase A and B, the solvent linear gradient procedure was set as: 50% A (0–3 min), 50–90% A (3–20 min), 90% A (20–29 min), 90–50% (29–32 min), and 50% A (32–35 min).

### Statistical Analysis

The data were analyzed by one-way analysis of variance (ANOVA) using the statistical software SPSS 17.0, and means were compared by Duncan’s multiple range test at 5%.

## Results

### Isolation and Identification of Lactic Acid Bacteria From Bacon Samples

**FIGURE 1:**
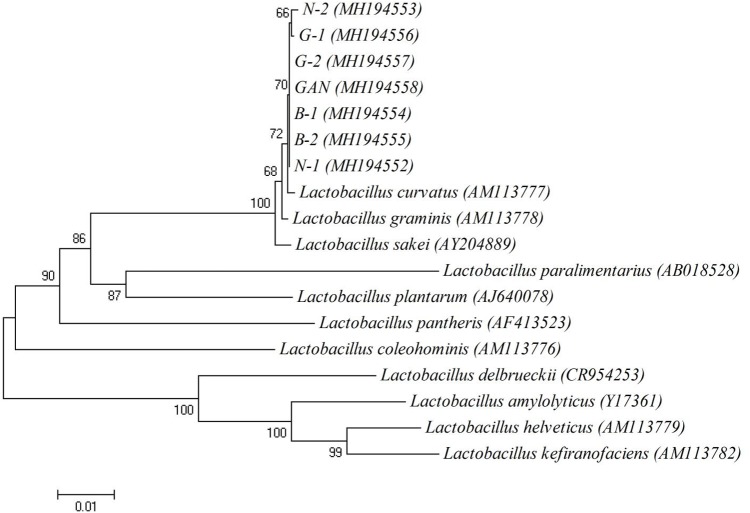
The phylogenetic tree based on 16S rDNA sequences. Numbers in parentheses represent accession numbers of 16S rDNA sequences of representative strains. The scale bar indicates 0.01 nucleotide substitution per position.

The typical Chinese bacon samples, including Sichuan bacon, Hunan bacon, Hubei bacon, Guangdong bacon, Shanxi, and Gansu bacon, were collected to isolate lactic acid bacteria. The BAs profiles of these samples were analyzed, and common six kinds of BAs were detected from different samples (Supplementary Table S1). All the samples showed the total BAs contents within the safe range, suggesting that the lactic acid bacteria in Chinese bacon may be conducive to control the BAs. CaCO_3_ can be dissolved by lactic acid bacteria to generate transparent zones, which is usually used to screen the candidates. Many clones showed the ability of dissolving calcium, and seven yellowish clones with big transparent zones were selected, named N-1, N-2, B-1, B-2, G-1, G-2, and GAN. The 16S rDNA fragments were amplified and sequenced to further identify the strains. Sequencing results revealed that the lengths of fragments were about 1468 bp, and all the sequences exhibited about 99% identity with that of the *L. curvatus* strains from the GenBank database (**Table 3**). The phylogenetic tree was shown in **Figure 1**, and as-selected seven strains were closely clustered with *L. curvatus*. The above results confirmed that these seven strains all belonged to *L. curvatus*, and corresponding 16S rDNA sequences were deposited in Genbank (accession numbers: MH194552, MH194553, MH194554, MH194555, MH194556, MH194557, and MH194558).

**Table 3:** 16S rDNA sequences similarities of isolated strains with representative lactic acid bacteria.

Isolates	Closest strains	Identities (%)	Accession no.
N-1	*L. curvatus* qz1219	99	LC129555
N-2	*L. curvatus* qz1220	99	LC129556
B-1	*L. curvatus* WiKim38	99	CP017124
B-2	*L. curvatus* MRS6	99	CP022474
G-1	*L. curvatus* WiKim52	99	CP016602
G-2	*L. curvatus* NRIC 0822	99	KM676454
GAN	*L. curvatus* NJ40	99	KJ914899

### Detection of BA-Producing Genes of the Strains

**FIGURE 2:**
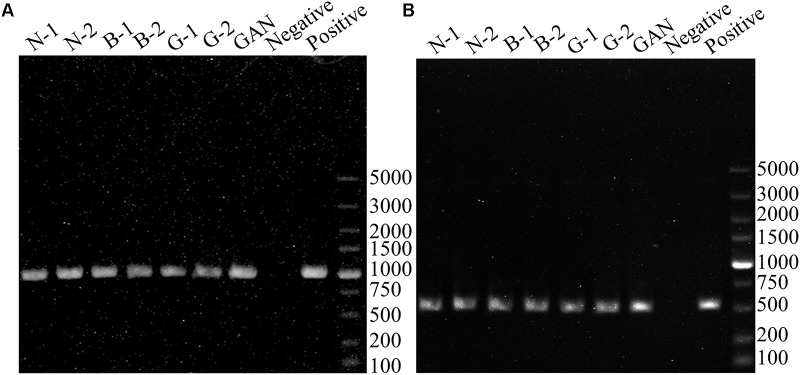
PCR results for the genes involved in BAs production. **(A)** PCR products using the primer pair TD2/TD5 for identification of tyrosine decarboxylase gene. **(B)** PCR products using the primer pair ODF/ODR for identification of ornithine decarboxylase gene. PCR positive controls (*Enterococcus faecium* DU-1 for tyrosine decarboxylase gene and *Oenococcus oeni* BJ for ornithine decarboxylase gene).

To evaluate the BAs-producing abilities of seven *L. curvatus* strains from the gene level, PCR assays were performed to verify the presence of amino acid decarboxylase genes (*hdcA*, *tyrdc*, *odc*, and *ldc*) and agmatine deiminase (*aguA* and *aguD*). None of the *L. curvatus* strains was positive in histidine decarboxylase gene (*hdcA*), agmatine deiminase gene (*aguA* and *aguD*), and lysine decarboxylase gene (*ldc*) screenings with the primers HdC1/HdC2, AgmSq1/AgmSq2, AgD1/AgD2, and Cad2F/Cad2R. These results indicated that the *L. curvatus* strains isolated might not have cadaverine and histamine formation ability, and they might not produce putrescine via agmatine. **Figure 2A** shows that all the *L. curvatus* strains were positive in tyrosine decarboxylase gene detection, and the resulting PCR amplicons were about 1100 bp (MH225426, MH225427, MH225428, MH225429, MH225430, MH225431, and MH225432), highly homologous (about 99%) with the *tyrdc* gene from *L. curvatus* HSCC1737 (AB086652). All the strains also indicated the positive results in detection of the ornithine decarboxylase gene (**Figure 2B**), and the fragments were about 500 bp (MH225433, MH225434, MH225435, MH225436, MH225437, MH225438, and MH225439), showing high similarity (about 80%) with the *odc* gene from *Oenococcus oeni* (FR751077). The above results revealed that all the *L. curvatus* strains might have the abilities of generating tyramine and putrescine.

### Evaluation of BAs-Producing Abilities of the Strains

The BAs-producing abilities of all strains were detected in MRS broth supplemented with corresponding precursors. After eliminating the BAs generated from MRS medium without precursors, four BAs were confirmed to be produced by all the *L. curvatus* strains, including tyramine, β-phenethylamine, putrescine, and cadaverine, and histamine and tryptamine were not detected. The dominant BA produced by *L. curvatus* strains was tyramine, followed by putrescine. Both ornithine and agmatine served as the precursors of putrescine, and the abilities of producing putrescine via ornithine were much higher than that of agmatine in our selected strains (**Table 4**). Additionally, all *L. curvatus* strains showed low cadaverine formation abilities. In comparison, the *L. curvatus* G-1 produced the lowest concentration of total BAs, especially the relative low level of tyramine and putrescine.

**Table 4:** The BA-producing abilities of the isolated strains with corresponding precursor.

Strain	Histamine (histidine; mg/L)	Tyramine (tyrosine; mg/L)	Tryptamine (tryptophan; mg/L)	β-Phenethylamine (phenylalanine; mg/L)	Putrescine (ornithine monohydrochloride; mg/L)	Putrescine (agmatine sulfate salt; mg/L)	Cadaverine (lysine; mg/L)	Total BAs (mg/L)
N-1	ND	717.31 ± 4.67 b	ND	55.88 ± 0.22 d	407.34 ± 2.74 c	0.90 ± 0.38 a	5.19 ± 0.48 b	1186.62 ± 8.49 b
N-2	ND	726.67 ± 0.98 c	ND	56.88 ± 2.27 d	417.63 ± 0.93 d	3.69 ± 0.06 b	7.71 ± 0.18 d	1212.58 ± 4.42 cd
B-1	ND	897.47 ± 1.88 f	ND	42.81 ± 2.13 a	470.84 ± 3.89 e	5.01 ± 0.18 c	10.94 ± 0.90 f	1427.07 ± 8.98 e
B-2	ND	739.38 ± 6.94 d	ND	55.48 ± 2.87 d	415.33 ± 1.22 d	6.33 ± 0.02 d	8.94 ± 0.14 e	1225.46 ± 11.19 d
G-1	ND	683.87 ± 2.28 a	ND	48.15 ± 0.58 b	380.04 ± 0.23 a	5.27 ± 1.14 c	6.08 ± 0.09 c	1123.41 ± 4.32 a
G-2	ND	727.34 ± 5.44 c	ND	51.99 ± 1.28 c	409.24 ± 1.22 c	6.22 ± 0.08 d	9.57 ± 0.06 e	1204.36 ± 8.08 c
GAN	ND	759.01 ± 1.22 e	ND	71.21 ± 0.32 e	385.60 ± 1.61 b	3.44 ± 0.13 b	4.11 ± 0.59 a	1223.46 ± 3.87 d

*Different letters (a, b, c, etc.) indicate significantly different means at *P* < 0.05 [analysis of variance (ANOVA)].*

### Detection of Gene Encoding Amines Degrading Enzyme

**FIGURE 3:**
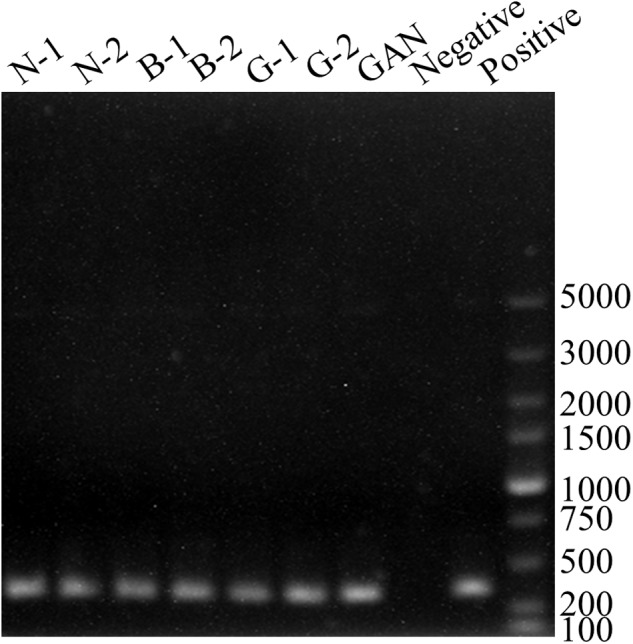
PCR results for genes involved in BAs degradation. PCR products using the primer pair BCf/BCr for identification of multi-copper oxidase gene. PCR positive control (*Lactobacillus fermenti* FJ for multi-copper oxidase gene).

In lactic acid bacteria, the multi-copper oxidase showed a high ability of degrading BAs (Guarcello et al., 2016). Therefore, the primers of BCf/BCr were used to detect the multi-copper oxidase gene. All strains exhibited positive results with BCf/BCr primer pair (**Figure 3**), and the lengths of corresponding PCR products were about 350 bp (MH225440, MH225441, MH225442, MH225443, MH225444, MH225445, and MH225446). Moreover, all PCR amplicons showed a high similarity above 95% with the multi-copper oxidase gene from *Lactobacillus paracasei* CB9CT (KU962939). Above results indicated all *L. curvatus* strains probably possessed the multi-copper oxidase for degrading BAs. Therefore, the BAs degradation abilities of all *L. curvatus* strains were further analyzed.

### Evaluation of the BAs Degradation Abilities of *L. curvatus*

The capacities of all *L. curvatus* strains to degrade BAs were assayed through the degradation test using the substrates of histamine, tyramine, tryptamine, β-phenethylamine, putrescine, and cadaverine. The BAs contents in broth and their corresponding controls were analyzed by HPLC. All the strains had the degradation abilities for each BA, especially for the tyramine, and different strains showed different degradation abilities (**Table 5**). In comparison, the *L. curvatus* G-1 demonstrated the highest degradation percentage, with the degradation efficiencies above 40% for all the BAs. In a whole, *L. curvatus* G-1 showed the highest BAs-degrading ability, as well as the lowest BAs-producing ability, and thus it exhibited the promising potential to control BAs.

**Table 5:** Profiles of BA-degradation rates of *L. curvatus* strains.

Strain	Histamine (%)	Tyramine (%)	Tryptamine (%)	β-Phenethylamine (%)	Putrescine (%)	Cadaverine (%)
N-1	6.94 ± 0.73 bcd	10.61 ± 3.78 a	6.99 ± 0.76 abc	6.40 ± 0.50 abc	8.04 ± 0.92 abc	7.14 ± 0.63 abc
N-2	7.96 ± 1.20 cd	15.64 ± 1.26 bc	8.88 ± 1.60 bc	7.56 ± 1.21 bc	9.39 ± 1.56 bc	8.37 ± 1.34 bc
B-1	9.46 ± 1.78 d	18.33 ± 4.35 c	9.17 ± 1.63 c	8.62 ± 1.58 c	10.44 ± 1.46 c	9.31 ± 1.38 bc
B-2	2.86 ± 1.26 a	17.63 ± 0.77 c	4.63 ± 1.67 a	3.93 ± 1.26 a	5.83 ± 0.25 a	4.63 ± 1.04 a
G-1	42.83 ± 2.50 e	42.98 ± 2.37 d	43.85 ± 1.95 d	41.50 ± 2.88 d	44.37 ± 2.36 d	43.64 ± 2.49 d
G-2	5.97 ± 1.20 bc	12.66 ± 0.04 ab	6.09 ± 1.62 ab	5.20 ± 1.56 ab	7.34 ± 1.66 ab	5.98 ± 1.56 ab
GAN	4.37 ± 1.08 ab	16.89 ± 1.38 bc	5.60 ± 1.76 a	4.22 ± 0.11 a	6.26 ± 0.62 a	5.00 ± 1.39 a

*Different letters (a, b, c, etc.) indicate significantly different means at P < 0.05 [analysis of variance (ANOVA)].*

### The BAs-Controlling Capacity of *L. curvatus* G-1 in Fermented Meat Model

**FIGURE 4:**
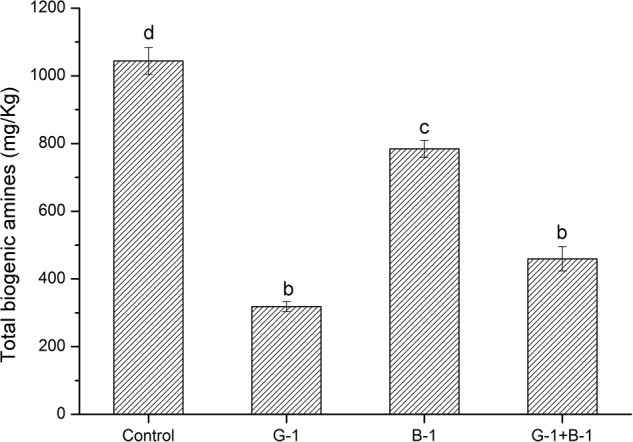
BAs contents in fermented meat product model inoculated with different strains. Different letters (a, b, c, etc.) indicate significantly different means at *P* < 0.05.

The *L. curvatus* G-1 might be the potential candidate for controlling the BAs due to its low BAs-producing ability and high BAs-degrading ability. Thus, we evaluated its BAs-controlling capacity in a fermented meat model. After culture at 37°C for 7 days, the total BAs contents of samples were analyzed and are shown in **Figure 4**. The control sample without inoculation of *L. curvatus* cells showed the highest total BAs content (1044.04 mg/kg), which was due to fermentation of native microbes in fresh meat. Inoculation with *L. curvatus* cells reduced the total BAs concentrations in each treatment. The lowest BAs concentration (318.19 mg/kg) was found in the sample inoculated with *L. curvatus* G-1, and it was much lower than that of *L. curvatus* B-1 (784.68 mg/kg), which agreed with their difference in BAs-producing and BAs-degrading abilities. The above results indicated that the *L. curvatus* G-1 had an excellent capacity for controlling BAs in fermented meat model.

## Discussion

The fermented meat products, usually processed from raw meat under open environment, undoubtedly consist of complex microorganisms, which are responsible for formation of good flavor and organoleptic characteristics. During the ripening of fermented meat products, proteins undergo degradation to generate large peptides, which are further hydrolyzed into oligopeptides and free amino acids (Landete et al., 2007a; Buňková et al., 2010). Some amino acids can be decarboxylated to BAs by microbes, which is undesirable for food safety. On the contrary, BAs can also be degraded by amino-oxidases of some microbes (Ladero et al., 2010). Lactic acid bacteria are usually used as starter cultures in meat products fermentation (Oki et al., 2011; Woraprayote et al., 2016), and they can produce lactic acid rapidly to decrease the pH of fermented meat product (Ladero et al., 2015), which can inhibit the growth of contaminant bacteria (Bover-Cid et al., 2001). Meanwhile, different lactic acid bacteria have different BAs formation and degradation abilities. Therefore, isolation of a lactic acid bacterium with low BAs formation and high BAs degradation ability is valuable to control the BAs in fermented meat products.

In order to screen a strain with low BAs formation and high BAs degradation ability, seven lactic acid bacteria were isolated from Chinese bacon. Based on 16S rDNA analysis, these seven strains were all identified as *L. curvatus*, which was reported as the predominant starter in some fermented meat products (Hugas et al., 1993; Freiding et al., 2011). Freiding et al. (2011) found that *L. curvatus* had no potential to produce histamine. Consistently, none of as-selected *L. curvatus* strains harbored histidine decarboxylase gene and produced histamine. Similar to previous reports (Freiding et al., 2011; Takebe et al., 2016), the tyrosine decarboxylase and ornithine decarboxylase genes were detected in as-selected *L. curvatus* strains, which agreed with the fact that all the strains could synthesize tyramine and putrescine. The enterococcal tyrosine decarboxylase was reported to be able to synthesize phenylethylamine from phenylalanine, which is structurally similar to tyrosine (Marcobal et al., 2006), and Landete et al. (2007b) also found that phenylethylamine was associated with tyramine production. Moreover, the *L. curvatus* strains were reported to be highly related to tyramine and β-phenylethylamine (Bover-Cid et al., 2001, 2008; De Las Rivas et al., 2008; Latorre-Moratalla et al., 2012, 2014; Li et al., 2012). Therefore, the phenylethylamine produced by *L. curvatus* strains isolated in this study may also result from the tyrosine decarboxylase. In addition, all isolated *L. curvatus* strains produced low level cadaverine, which is consistent with a previous study (Bover-Cid et al., 2008), while the lysine decarboxylase genes were not detected in all *L. curvatus* strains. Similarly, low level of putrescine was produced via agmatine, while agmatine deiminase genes were also negative in all isolated strains. These results indicate that unknown lysine decarboxylase or agmatine deiminase with low activities may exist in *L. curvatus*, which will be further investigated in the future. Suzzi and Gardini (2003) reported that the activities of amino acid decarboxylases were usually strain-specific. Among the seven strains, *L. curvatus* G-1 shows the lowest BAs-producing ability, which may be due to its low amino acid decarboxylase activity.

Bacteria with amine oxidase have the potential to reduce BAs in fermented meat products (Zaman et al., 2011). The amine oxidases also exist in lactic acid bacteria (Callejón et al., 2014; Guarcello et al., 2016). In a previous study, the multi-copper oxidase in lactic acid bacteria showed the potent ability to degrade BAs (Ladero et al., 2014), and *L. curvatus* was also reported to reduce the BAs in fermented vegetable and fermented sausage (Rabie et al., 2011; Mangia et al., 2013; Kim et al., 2017). Herein, all the *L. curvatus* strains isolated were confirmed to harbor multi-copper oxidase genes, and the sequences obtained were highly similar to that of *L. paracasei* CB9CT (Guarcello et al., 2016). The BAs degradation test indicated that all the *L. curvatus* strains isolated could degrade six common BAs. In comparison, the BAs-degrading capacity of *L. curvatus* G-1 was much higher than other strains. Therefore, *L. curvatus* G-1 was further assessed in a fermented meat product model, and our results show that *L. curvatus* G-1 can be used as starter cultures for reducing BAs in fermented meat product.

## Conclusion

This study obtains seven *L. curvatus* strains from Chinese bacon, and they possess different BAs-producing and degrading abilities. Importantly, the *L. curvatus* G-1 not only indicates a relative low BAs-producing ability, but also exhibits a high BAs-degrading activity. Moreover, *L. curvatus* G-1 can efficiently control the BAs in the fermented meat model. This study provides a promising BAs-controlling strain of *L. curvatus* G-1, which will be further investigated as the starter cultures in the practical production of fermented meat products.

## Author Contributions

XuW, LW, and LL designed the study. XiW and LL executed the experimental work and analyzed the data. XuW and LW contributed reagents and materials. LL, XuW, ZW, SC, and LW wrote and revised the manuscript. All authors read and approved the final manuscript.

## Conflict of Interest Statement

The authors declare that the research was conducted in the absence of any commercial or financial relationships that could be construed as a potential conflict of interest.

## Footnotes

1 http://blast.ncbi.nlm.nih.gov/Blast.cgi

2 http://blast.ncbi.nlm.nih.gov/Blast.cgi
