# Supplementary material for: Evaluation of the Biogenic Amines Formation and Degradation Abilities of Lactobacillus curvatus From Chinese Bacon
Source: Front Microbiol. 2018 May 15;9:1015. doi: 10.3389/fmicb.2018.01015 (PMC5962796; doi:10.3389/fmicb.2018.01015)
Supplement: Supplementary file 1 [file Data_Sheet_1.DOCX]

Supplementary Materials

Table S1 Contents of BAs in bacon samples.

| Bacon  sample | Histamine (mg/Kg) | Tyramine (mg/Kg) | Tryptamine (mg/Kg) | β-Phenethylamine (mg/Kg) | Putrescine (mg/Kg) | Cadaverine (mg/Kg) | Total  (mg/Kg) |
| --- | --- | --- | --- | --- | --- | --- | --- |
| Sichuan | ND | 115.61±4.53 b | 6.71±1.74 | ND | 16.51±2.12 b | 26.46±1.84 b | 165.29±10.23 b |
| Hunan | ND | 150.54±9.39 c | ND | 24.06±1.75 b | 27.78±0.43 d | 25.98±3.02 b | 228.36±14.59 c |
| Hubei | ND | 18.63±2.61 a | ND | 3.01±0.43 a | 2.02±0.92 a | 33.34±3.77 c | 57.00±7.73 a |
| Guangdong | ND | 177.47±11.66 d | ND | 27.13±1.92 b | 24.64±0.93 c | 29.07±3.93 bc | 258.31±18.44 d |
| Shanxi | 1.19±0.05 | 19.11±0.97 a | ND | 1.21±0.50 a | 2.57±0.12 a | 20.20±0.55 a | 44.28±2.19 a |
| Gansu | ND | 198.80±8.77 e | ND | 34.92±7.55 c | 28.26±1.65 d | 25.93±1.27 b | 287.91±19.24 e |

Different letters (a, b, c, etc.) indicate significantly different means at P < 0.05 (analysis of variance (ANOVA)).
